# Does the use of AI tools in academic publisinng represent a new form of ghost authorship?

**DOI:** 10.3325/cmj.2023.64.292

**Published:** 2023-08

To the Editor: I read with great interest the article *Exploring the adoption of ChatGPT in academic publishing: insights and lessons for scientific writing* by Jan Homolak (1). The author elegantly explained the advantages and drawbacks of using artificial intelligence (AI) in scientific writing and outlined the ethical dilemma posed by the use of ChatGPT and other AI tools in the field (1).

After the author ran 200 published abstracts through the ZeroGPT AI-detection tool, 59.5% and 79.2% of original and review article abstracts, respectively, showed the presence of AI-generated content. The findings raise a concern as to whether the use of AI in academic writing, if human authors do not declare such use, represents a new form of ghost authorship (2,3). This kind of use creates unequal opportunities among authors, especially when the AI-created content cannot be detected by available AI detection tools (4).

The author repeated the evaluation using different AI-detecton tools, namely the OpenAI classifier and GPTZero; however, they showed lower scores than ZeroGPT. In a further step to affirm the reliability of the three tools in detecting AI-generated text, the author evaluated abstracts published before the AI era, showing a high rate of false-positive results (1).

Another major concern is related to the ability of ChatGPT to create full abstracts even in the absence of complete research data. In such a situation, the trustworthiness of abstracts submitted to scientific conferences is seriously compromised (5).

Finally, I agree with Dr Homolak that AI in scientific writing is here to stay, but how should its role be regulated? Should it be considered an author of a scientific paper? Will there be a parallel AI peer-review system? Moreover, will journals create a specific section for articles written with the help of AI? I believe these questions and other emerging concerns must be promptly addressed before AI is widely adopted in scientific research and writing.
